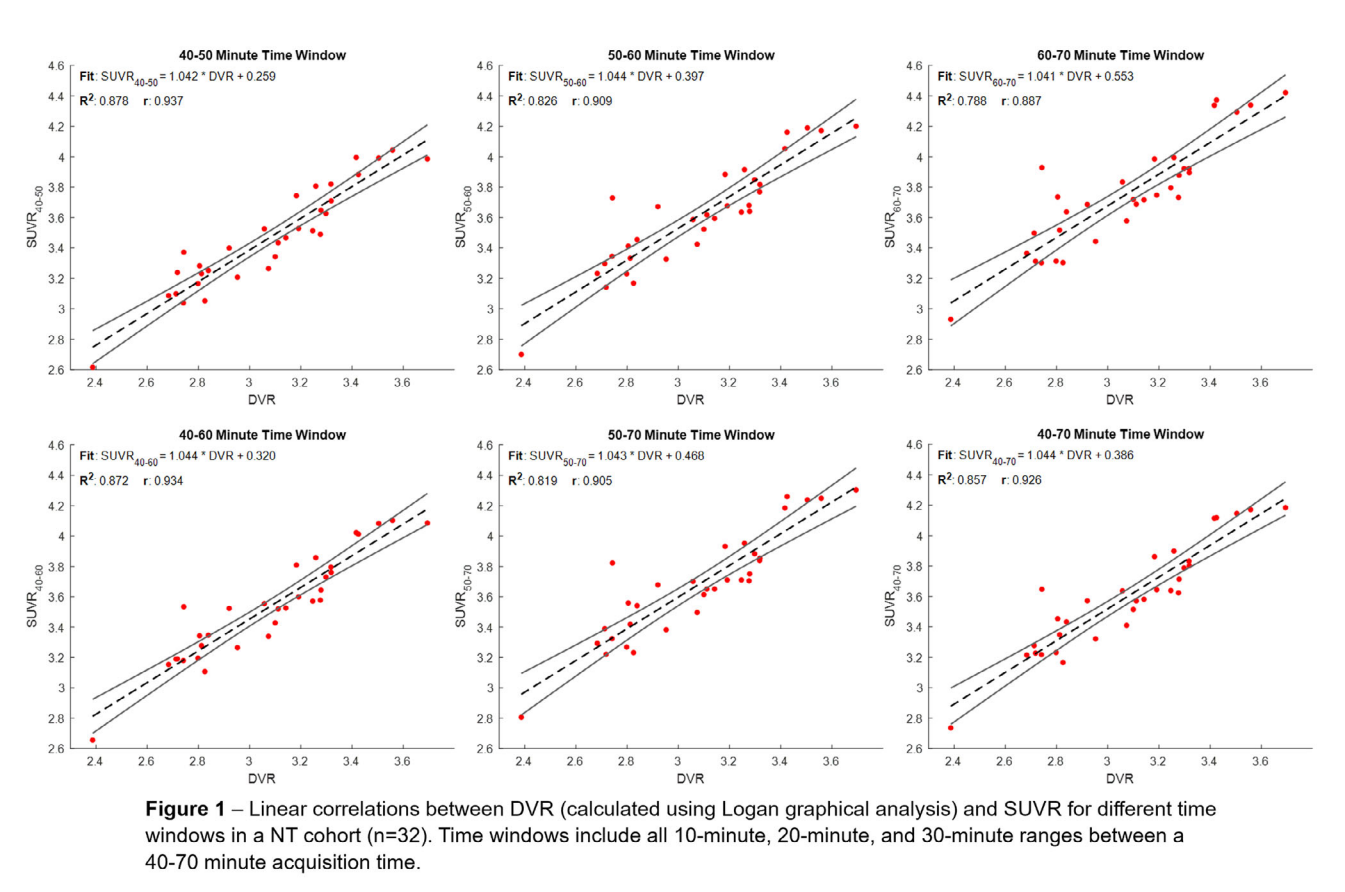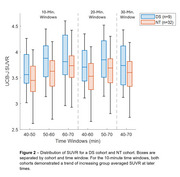# Relationship between tau and SV2A in the neurotypical population and translation to the Down syndrome population

**DOI:** 10.1002/alz70856_103321

**Published:** 2025-12-25

**Authors:** Max McLachlan, Alexandra H DiFilippo, Brecca Bettcher, Lisette LeMerise, Andrew K McVea, Matthew D Zammit, Sigan L Hartley, Barbara B. Bendlin, Bradley T Christian

**Affiliations:** ^1^ Waisman Center, University of Wisconsin‐Madison, Madison, WI, USA; ^2^ Wisconsin Alzheimer's Disease Research Center, School of Medicine and Public Health, University of Wisconsin‐Madison, Madison, WI, USA

## Abstract

**Background:**

Synaptic density loss has been proposed as a major neuronal mechanism through which cognition degrades in Alzheimer's disease (AD). PET imaging studies have observed synaptic density change across the AD continuum using SV2A radiotracer [^11^C]UCB‐J. Recent work from the SYNAPSE study revealed strong associations between neurofibrillary tau burden and reduced synaptic density in older adults (DiFilippo, under review). These analyses are actively being extended to the Down syndrome (DS) population. Individuals with DS carry a genetic risk for AD, resulting in early and rapid accumulation of AD pathology. In the Alzheimer Biomarkers Consortium – Down syndrome (ABC‐DS) study, [^11^C]UCB‐J PET imaging is underway. This project will characterize synaptic density relative to the accelerated accumulation of AD neuropathology in DS.

**Method:**

[^11^C]UCB‐J PET analysis techniques require validation before extending their use to the DS population. Brain morphology differences in adults with DS limit the efficacy of spatial processing algorithms designed for the neurotypical (NT) population. Therefore, DS‐specific brain templates for T1 MR and [^11^C]UCB‐J PET images will be created. Additionally, kinetic modelling has not been performed with UCB‐J in the DS population, and only late‐time window uptake ratios (i.e. SUVR) will be quantified to minimize experimental burden. To evaluate SUVR PET methods in the DS cohort, equivalent SUVR methods will be explored in a NT cohort, using DVR as a reference to determine optimal windowing parameters. The relationship between regional tau and SV2A can then be investigated in both the NT and DS populations.

**Result:**

A DS‐specific T1 MRI template has been developed (LeMerise; 2022), and a [^11^C]UCB‐J PET template is being created. SUVR windowing for 40‐70 minute acquisitions was compared to DVR in a NT cohort (Figure 1) and compared with the same parameters in a DS cohort (Figure 2). In a separate NT cohort (*n* = 94; age=56.3‐89.8 years), a strong relationship between hippocampal tau and SV2A was observed (r=‐0.49, *p* <0.001), with a weaker association in the entorhinal cortex (r=‐0.24, *p* = 0.02). Tau analyses are actively being performed for the DS population.

**Conclusion:**

Understanding the distribution of SV2A in DS will provide insight on changes to synaptic density in populations with accelerated AD neuropathology.